# Discovery of HCD3514 as a potent EGFR inhibitor against C797S mutation *in vitro* and *in vivo*

**DOI:** 10.7150/jca.77788

**Published:** 2023-01-01

**Authors:** Mengzhen Lai, Tao Zhang, Hao Chen, Peiran Song, Linjiang Tong, Jiaying Chen, Yingqiang Liu, Yi Ning, Fang Feng, Yan Li, Haotian Tang, Yi Chen, Yan Fang, Xiaoyun Lu, Meiyu Geng, Ke Ding, Ker Yu, Jian Ding, Hua Xie

**Affiliations:** 1 Department of Pharmacology, School of Pharmacy, Fudan University, Shanghai 201203, China.; 2 Division of Antitumor Pharmacology, State Key Laboratory of Drug Research, Shanghai Institute of Materia Medica, Chinese Academy of Sciences, Shanghai 201203, China.; 3 International Cooperative Laboratory of Traditional Chinese Medicine Modernization and Innovative Drug Development of Chinese Ministry of Education (MOE), Guangzhou City Key Laboratory of Precision Chemical Drug Development, School of Pharmacy, Jinan University, Guangzhou 510632, China.; 4 Zhongshan Institute for Drug Discovery, Shanghai Institute of Materia Medica, Chinese Academy of Sciences, Zhongshan 528400, China.; 5 Hangzhou Institute for Advanced Study, University of Chinese Academy of Sciences, Hangzhou 310024, China.; 6 University of Chinese Academy of Sciences, Beijing 100049, China.

**Keywords:** fourth-generation EGFR inhibitor, EGFR^C797S^ mutation, NSCLC, drug resistance, small molecular inhibitor

## Abstract

Osimertinib (AZD9291), a third-generation epidermal growth factor receptor (EGFR) tyrosine kinase inhibitors (TKI), has significantly improved the survival of non-small cell lung cancer (NSCLC) patients with EGFR^T790M^ mutation, the major mechanism of acquired resistance to first-generation EGFR TKI. However, resistance to AZD9291 arises eventually and EGFR^C797S^ mutation was reported to be a major resistance mechanism. Thus, it is highly valuable to develop novel EGFR fourth-generation inhibitors targeting C797S mutation to override the acquired resistance. In this study, we identified HCD3514 as a novel EGFR fourth-generation inhibitors targeting C797S triple mutation. It strongly inhibited EGFR^L858R/T790M/C797S^ and EGFR^19del/T790M/C797S^ mutations with IC_50_ values of 1.0 and 2.0 nM, respectively. HCD3514 dose-dependently inhibited the activation of EGFR in both engineered BaF3 cells and tumor cells harboring EGFR^C797S^ triple mutant and thus effectively suppressed the proliferation of the cells. Moreover, HCD3514 caused a dose-dependent increase of apoptosis in C797S triple mutant cells accompanied by increased levels of cleaved caspase-3 and cleaved PARP. Furthermore, HCD3514 induced tumor growth inhibition in EGFR^19del/T790M/C797S^ xenograft model as a single oral agent by decreasing the activation of EGFR. In addition to EGFR^C797S^ triple mutations, HCD3514 also potently and selectively inhibited EGFR^T790M^ double mutations (L858R/T790M and 19del/T790M). Collectively, HCD3514 is a highly selective and potent EGFR inhibitor against EGFR^C797S^ triple mutations as well as EGFR^T790M^ double mutations and is confirmed potently anti-tumor activity in preclinical models.

## Introduction

Lung cancer is a malignant tumor with high incidence and mortality throughout the world, being responsible for almost one-quarter of all cancer-related deaths 1. The epidermal growth factor receptor (EGFR) is one of the most important driver oncogenes in lung cancer, especially non-small cell lung cancer (NSCLC), which accounts for about 85% of lung cancer 2, 3. Diverse activating mutations in EGFR occur in 10% to 50% of patients with NSCLC 4, 5. The two most common EGFR activating mutations are the small in-frame deletions in exon 19 (19del) and L858R point mutation in exon 21, occupying more than 90% of all EGFR mutations 6, 7. NSCLC patients harboring activating mutations respond well to the first-generation EGFR tyrosine kinase inhibitors (TKIs), including gefitinib, erlotinib and icotinib 8-10. But acquired resistance arises within 9-15 months after the treatment of first-generation EGFR TKIs 11. The secondary T790M mutation in EGFR exon 20 is the primary resistance mechanism, contributing to approximately 60% of resistance cases 12, 13. Hence, the second-generation EGFR TKI such as afatinib has been developed, but it still fails to overcome resistance due to the poor selectivity over wild-type EGFR (EGFR^WT^), resulting in severe side effects 14, 15.

Consequently, the urgent need for highly selective third-generation EGFR TKIs that target both activating mutations and T790M resistance mutation leads to the development of osimertinib (AZD9291). AZD9291 exhibits remarkable efficacy in treating patients of T790M mutation-positive NSCLC 16, 17. More recently, third-generation EGFR TKIs, including almonertinib and furmonertinib have been approved to target mutant EGFR bearing T790M 18, 19. However, resistance inevitably emerges in most patients. The acquired resistance mechanisms to AZD9291 are complicated, among which C797S mutation is the most common on-target resistance mechanism 20. C797S mutation occurs in EGFR exon 20 and accounts for 10-26% of resistant cases to AZD9291 as second-line therapy 21-23. Therefore, the discovery of fourth-generation EGFR inhibitors that can effectively inhibit EGFR^T790M/C797S^ resistance mutation has gained much attention.

Currently, encouraging achievements have been made in the development of the fourth-generation EGFR TKIs, which could be summarized into two categories based on their discovery strategies. 1) Exploiting allosteric binding pocket that is remote from the location of C797S mutation. For example, EAI045, an allosteric inhibitor, is reported as the first fourth-generation EGFR inhibitor overcoming T790M and C797S resistance mutation 24. However, EAI045 is not effective in the absence of combination with anti-EGFR antibody such as cetuximab. Subsequently, its derivative JBJ-04-125-02 exhibits enhanced efficacy as a single agent but it is not effective to overcome 19del/T790M/C797S mutation 25. 2) Developing ATP-competitive inhibitors through structural modification of ALK/EGFR dual inhibitor brigatinib or the third-generation EGFR TKIs. Brigatinib is reported to be potent against EGFR triple mutation *in vitro* and *in vivo*, but quite similar to that with EAI045 treatment, its antitumor efficacy is limited as a single agent 26. Further potency improvement-oriented modification of brigatinib leads to the discovery of a series of EGFR^T790M/C797S^ inhibitors showing potential antitumor activity of AZD9291-resistant triple mutant EGFR *in vitro* and *in vivo*, including TQB3804, LS-106 27 and compound 12 28. Besides, modification based on scaffolds of third-generation EGFR TKIs is proposed to maintain inhibitory potency and mutant selectivity against EGFR^T790M^. Typical examples include BI-4020 29 (from EGF-816 30), CH7233163 31 (from compound 42 32) and JND3229 33/compound 8r-B 34 (from compound 2v 35). We also reported a series of macrocyclic compounds based on the structure of AZD9291 36. Nonetheless, up to the present, there are still no fourth-generation EGFR inhibitor approved for C797S-resistant patients with disease progression following treatment with AZD9291, it is necessary to develop novel fourth-generation EGFR TKIs.

In this study, through structural hybridization of brigatinib with EGFR^T790M^ inhibitor AZD9291, we identified a potent fourth-generation EGFR inhibitor, HCD3514, which is effective as a single agent to overcome EGFR triple mutation (EGFR^L858R/T790M/C797S^ and EGFR^19del/T790M/C797S^) and shows promising antitumor activity both *in vitro* and *in vivo*.

## Materials and Methods

### Cell culture

BaF3 cells and PC-9 cells were obtained from Deutsche Sammlung von Mikroorganismen und Zellkulturen GmbH (DSMZ) and the European Collection of Authenticated Cell Cultures (ECACC), respectively. MRC-9 cells and GSE-1 cells were purchased from the American Type Culture Collection (ATCC). All the cells were maintained in RPMI-1640 medium (Gibco) or EMEM medium (Gibco) supplemented with 10% FBS (Gibco) at 37 °C in 5% CO_2_.

BaF3 cells were transduced with retroviruses harboring genes encoding EGFR^L858R/T790M/C797S^, EGFR^19del/T790M/C797S^, EGFR^L858R/T790M^ or EGFR^19del/T790M^ and cells stably expressing these mutants were subsequently selected in medium supplemented with 1 μg/mL puromycin (Sigma-Aldrich). PC-9-OR cells harboring EGFR^19del/T790M/C797S^ mutation were constructed by CRISPR/Cas9 genome-editing technology to simultaneously knock-in the T790M and C797S mutations into PC-9 cells harboring EGFR^19del^ 27. All cells in the study were authenticated by short tandem repeat (STR) analysis performed by Genesky.

### Compounds

Compound HCD3514 was designed and synthesized by Ke Ding laboratory. ^1^H NMR and ^13^C NMR spectra was recorded on a Bruker AV-400 spectrometer at 400 MHz and Bruker AV-600 spectrometer at 151 MHz, respectively, in CDCl_3_. Coupling constants (*J*) are expressed in hertz (Hz). Chemical shifts (δ) of NMR are reported in parts per million (ppm) units relative to internal control (TMS). The first-order peak patterns are indicated as *s* (singlet), *d* (doublet), *t* (triplet), *q* (quadruplet). Complex non-first order signals are indicated as *m* (multiplet). The high-resolution ESI-MS results were recorded on an Applied Biosystems Q-STAR Elite ESI LC-MS/MS mass spectrometer. The purity of compound was determined by reverse-phase high-performance liquid chromatography (HPLC) analysis using an Agilent 1260 system (G1310B Iso pump and G1365D MWD VL detector) with an YMC Triart C18 reversed-phase column (250 mm × 4.6 mm, 5 μm) at 254 nm. Elution was MeOH in water, and flow rate was 1.0 mL/min.^1^H NMR (400 MHz, CDCl_3_) δ 8.43 (s, 1H), 8.41 (s, 1H), 8.35 (d, *J* = 8.0 Hz, 1H), 7.96 (d, *J* = 8.3 Hz, 1H), 7.43 (t, *J* = 7.7 Hz, 1H), 7.33 (t, *J* = 7.6 Hz, 2H), 7.24 (s, 1H), 7.06 (d, *J* = 8.4 Hz, 1H), 6.87 (d, *J* = 8.4 Hz, 1H), 4.58 - 4.42 (m, 1H), 3.60 - 3.53 (m, 2H), 3.41 (q, *J* = 7.3 Hz, 2H), 2.79 - 2.63 (m, 4H), 2.62 - 2.43 (m, 6H), 2.43 - 2.36 (m, 1H), 2.34 (s, 3H), 1.99 - 1.90 (m, 2H), 1.79 - 1.67 (m, 2H), 1.28 (t, *J* = 5.9 Hz, 3H), 1.26 (s, 3H), 1.24 (s, 3H).13C NMR (151 MHz, CDCl_3_) δ 158.43, 158.22, 156.93, 150.71, 139.15, 134.79, 133.85, 130.39, 128.78, 125.49, 124.13, 123.55, 118.64, 117.55, 116.60, 113.01, 112.83, 108.99, 70.38, 61.96, 55.46 (2C), 50.81 (2C), 49.13, 48.94, 46.05, 28.80 (2C), 22.18 (2C), 8.14, 1.05. HRMS (m/z): [M+H] + calculated for C33H42ClN7O3S, 652.2831; found, 652.2814. HPLC purity: 98.1%. Brigatinib (cat. no. S8229) and AZD9291 (cat. no. S7297) were purchased from Selleck Chemicals.

### *In vitro* kinase assay

The recombinant EGFR proteins of the kinase domain were purchased from SignalChem Lifesciences (EGFR^L858R/T790M/C797S^: cat. no. E10-12VG, EGFR^19del/T790M/C797S^: cat. no. E10-12UG, EGFR^L858R/T790M^: cat. no. E10-122DG, EGFR^19del/T790M^: cat. no. E10-122KG) or Eurofins Scientific (EGFR^WT^: cat. no. 14-531M). ELISA was used to evaluate the inhibitory activity of test compounds against EGFR, and the experiment was performed as described previously 37.

### Molecular docking

The molecular docking procedure was performed within Glide 7.9 software 38, and the results were prepared by PyMol 2.6 software. The protein-ligand complex crystal structure of AZD9291 with EGFR^L858R/T790M/C797S^ was selected as the template to elucidate the binding mode of HCD3514. Protein structure was downloaded from Protein Data Bank (PDB 6LUD). The EGFR enzyme was defined as a receptor and the docking grid was centered on the ligand binding location. Docking simulations were performed in standard precision (SP) mode. Other parameters were set as default. After accomplishment of the molecular docking procedure, eight docking poses were scored and selected based on calculated energy.

### Cell proliferation assay

BaF3 cells, PC-9-OR cells, MRC-9 cells and GSE-1 cells were plated in 96-well plates in the corresponding medium and incubated overnight, followed by exposing to medium containing serial dilutions of compounds (HCD3514, brigatinib and AZD9291). After 72 h of drug treatment, cell counting kit-8 (CCK8) or sulforhodamine B (SRB) was added to each well and then measured by SoftMax Pro software at the absorbance of 450 nm or 560 nm 39.

### Western blot analysis and antibodies

Western blotting was performed as described previously 40. Cells were collected by trypsin and then lysed with SDS lysis buffer. Tumor tissues were homogenized in RIPA buffer on ice, followed by centrifugation at 12,000 x g for 30 min. The concentration of protein was measured with BCA protein assay kit (cat. no. 23227, Thermo Fisher Scientific). After heating at 100 °C for 15 min, protein samples were separated on 8-12% SDS-PAGE gels, and then transferred to nitrocellulose membranes (Life Technologies). Membranes were blocked in 5% nonfat milk-TBST and immunoblotted with primary antibodies against phospho-EGFR (Tyr1068; dilution, 1:500; cat. no. 2234L, CST), EGFR (dilution, 1:1000; cat. no. 4267S, CST), PARP (dilution, 1:1000; cat. no. 9542S, CST), caspase-3 (dilution, 1:1000; cat. no. 9662S, CST), cleaved caspase-3 (dilution, 1:1000; cat. no. 9664S, CST), phospho-Erk (Thr202/Tyr204; dilution, 1:1000; cat. no. 4370S, CST), Erk (dilution, 1:1000; cat. no. 4695S, CST) and β-actin (dilution, 1:20000; cat. no. 60008-1-Ig, Proteintech) at 4 °C overnight and then incubated with secondary antibodies (cat. no. 111-035-003, Jackson).

### Cell apoptosis analysis

BaF3-EGFR^L858R/T790M/C797S^ cells, BaF3-EGFR^19del/T790M/C797S^ cells and PC-9-OR cells were seeded in 6-wells plates overnight, and exposed to indicated concentrations of compounds HCD3514 or brigatinib for 48 h. Then cells were collected and washed with PBS and measured by Annexin V-FITC Apoptosis detection kit (cat. no. A211-02, Vazyme Biotechnology). Signals were measured using FACS Calibur flow cytometer, and FlowJo software was used to analyze the data.

### Animal study

BaF3-EGFR^19del/T790M/C797S^ cells (2 × 10^6^) were injected subcutaneously into BALB/c nude mice (Shanghai institute of medicine). After animals were randomly assigned to groups, tumor-bearing mice were orally administered vehicle, test compound HCD3514 or AZD9291 at the indicated doses once a day and tumor size were measured once per week. Tumor volume was calculated as length × width^2^ × 0.5 (mm^3^) and the tumor growth inhibition (TGI) = [1-RTV (treated) / RTV (control)] × 100%. Mice were sacrificed and tumors were harvested for Western blot analysis. All animal study was approved by the Institutional Animal Care and Use Committee of the Shanghai Institute of Materia Medica and strictly performed according to the institutional ethical guidelines on animal care.

### Statistical analysis

Data were presented as mean ± Standard Deviation (SD) and were analyzed by GraphPad Prism 8.0 software. Statistical analysis of the difference between vehicle and compound treated groups was compared by a two-tailed Student's t-test. Statistical significance was defined as *P < 0.05, **P < 0.01, ***P < 0.001.

## Results

### Identification of HCD3514 as a selective inhibitor of EGFR^C797S^ triple mutant

**Figure 1:**
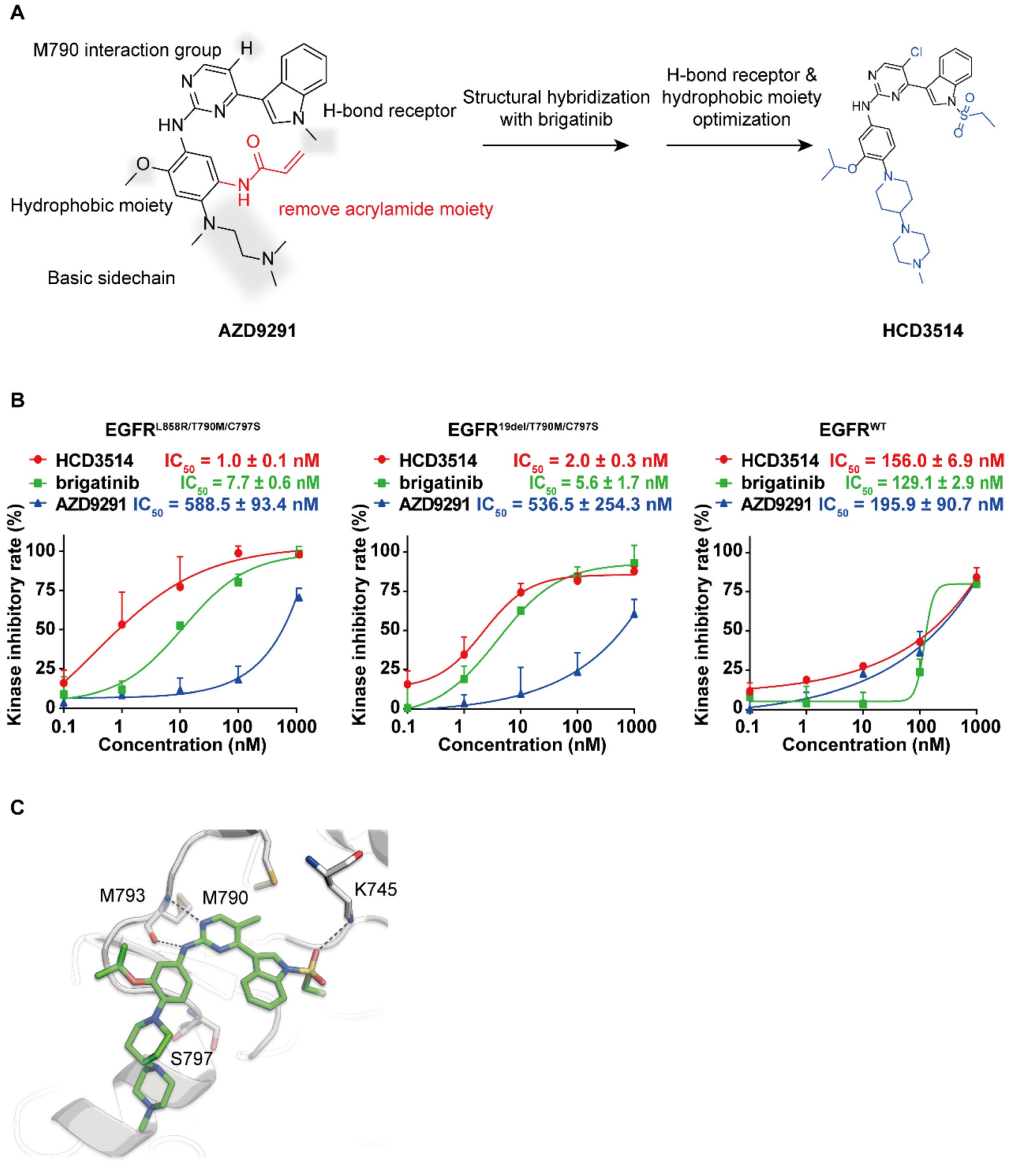
** The synthesis and kinase inhibitory activities of HCD3514. (A)** Design and synthesis of compound HCD3514. **(B)** Kinase inhibitory activities of HCD3514, brigatinib and AZD9291 against EGFR^L858R/T790M/C797S^, EGFR^19del/T790M/C797S^ and EGFR^WT^. The IC_50_ values were shown as mean ± SD of three independent experiments. **(C)** The docking structure of HCD3514 with EGFR^L858R/T790M/C797S^ (protein from PDB ID: 6LUD).

To obtain a novel and selective inhibitor targeting EGFR^T790M/C797S^, we incorporated important pharmacophore of brigatinib to the 4-indolyl-2-phenylaminopyrimidine scaffold of EGFR^T790M^ inhibitor AZD9291 and developed a novel series of potent and mutant selective EGFR^T790M/C797S^ inhibitor. Following extensive medicinal chemistry optimization by adding H-bond receptor of the indole ring and hydrophobic moiety on the benzene ring, we identified HCD3514 as a selective EGFR^T790M/C797S^ inhibitor (Figure 1A).

ELISA assay was used to determine the kinase inhibitory activities of HCD3514. Brigatinib and AZD9291 were used as positive and negative control compounds, respectively. HCD3514 exhibited nanomolar potency against EGFR^L858R/T790M/C797S^ (IC_50_ = 1.0 ± 0.1 nM) and EGFR^19del/T790M/C797S^ (IC_50_ = 2.0 ± 0.3 nM) with a more than 78-fold selectivity over EGFR^WT^ (IC_50_ = 156.0 ± 6.9 nM). By comparison, HCD3514 possessed more potent kinase inhibitory activity against EGFR triple mutations than brigatinib (Figure 1B).

To further elucidate the binding interaction of HCD3514 and EGFR^L858R/T790M/C797S^, a molecular docking experiment was performed. As illustrated in Fig 1C, HCD3514 bound to the ATP binding site of EGFR with a reversible "U-shaped" configuration similar to AZD9291. The aminopyrimidine core formed a bidentate hydrogen bond interaction with the “hinge” residue Met793 and the chloride atom was directed toward the mutated Met790. Notably, an additional hydrogen bond was observed between sulfonate oxygen and Lys745, which may explain the high potency against EGFR^C797S^ mutants.

### Antiproliferative activity and target inhibition of HCD3514 in EGFR^C797S^ triple mutant cells

**Figure 2:**
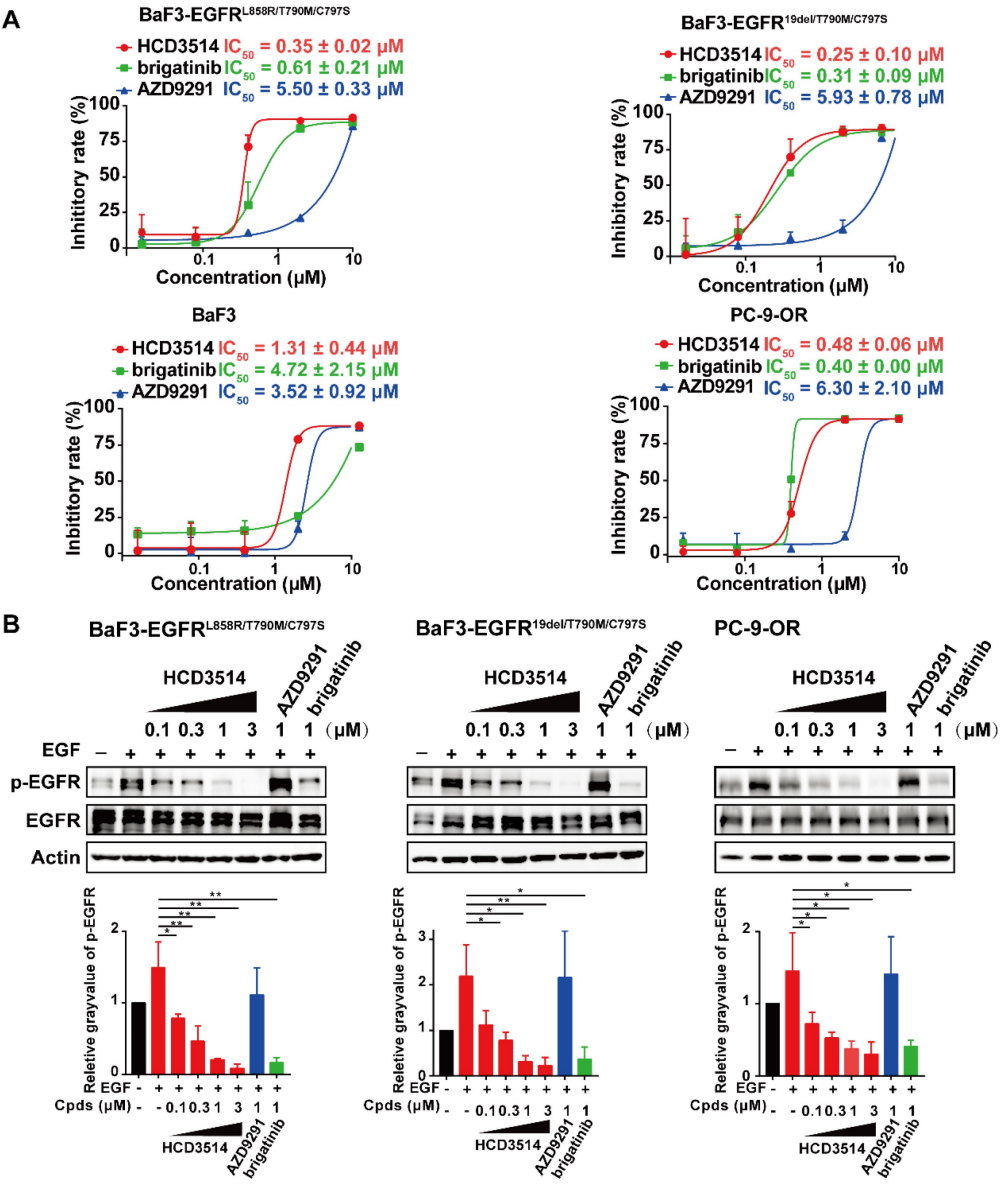
** Cellular activity of HCD3514 against engineered-BaF3 cells and PC-9-OR tumor cells expressing EGFR^C797S^ triple mutation. (A)** Antiproliferation activity of HCD3514, brigatinib and AZD9291 in BaF3 parental cells, BaF3 cells that overexpressed EGFR^L858R/T790M/C797S^ or EGFR^19del /T790M/C797S^ triple mutation, as well as PC-9-OR cells. The IC_50_ values were shown as mean ± SD of three independent experiments. **(B)** The activities of the compounds on the activation of EGFR mutant in these cells. All cells were treated with HCD3514, brigatinib or AZD9291 at indicated concentration for 2 h, and the expression of phospho-EGFR and total EGFR was detected. The phospho-EGFR expression was quantified by ImageJ software and data shown as mean ± SD of three independent experiments. *P < 0.05, **P < 0.01.

We next investigated the cellular inhibitory activity of HCD3514 in BaF3 cells overexpressing EGFR^L858R/T790M/C797S^ or EGFR^19del/T790M/C797S^ triple mutations, respectively. HCD3514 potently inhibited the proliferation of BaF3-EGFR^L858R/T790M/C797S^ and BaF3-EGFR^19del/T790M/C797S^ cells with IC_50_ of 0.35 μM and 0.25 μM, respectively, while BaF3 parental cells were less responsive to HCD3514 with IC_50_ of 1.31 μM. Similar results were observed in the treatment with brigatinib, which selectively inhibited the proliferation of the two EGFR triple mutant BaF3 cells. Whereas AZD9291 showed less potent inhibition of proliferation against both C797S mutant cells. Besides, we also constructed PC-9-OR tumor cells 27 expressing EGFR^19del/T790M/C797S^ mutations using CRISPR/Cas9 knock-in technology in PC-9 cells, an EGFR^19del^-mutant lung cancer cell line. HCD3514 also displayed an excellent antiproliferative potency in PC-9-OR cells, with IC_50_ of 0.48 μM, which was similar to that of brigatinib (IC_50_ = 0.40 μM) and more potent than AZD9291 (IC_50_ = 6.30 μM) (Figure 2A).

To further verify the mechanism of the antiproliferative activity, Western blot assay was employed to examine the ability of HCD3514 to inhibit cellular EGFR phosphorylation. The results revealed that HCD3514 potently and dose-dependently suppressed the EGFR phosphorylation in both triple mutant BaF3 cells and PC-9-OR cells (Figure 2B). Protein expression of phosphorylated EGFR was significantly reduced following brigatinib treatment, but AZD9291 at 1 μM concentration failed to suppress the level of EGFR phosphorylation in all three EGFR^C797S^ triple mutant cells.

Moreover, to evaluate the cytotoxic effect of HCD3514, we also tested the antiproliferative activity of HCD3514 in normal human lung (MRC-9) and gastric mucosal (GSE-1) cell lines. As shown in Supplementary Figure S1, both the human normal cell lines (MRC-9 and GSE-1 cells) were less responsive and showed weak growth inhibition after HCD3514 treatment with IC_50_ of 1.03 μM and 1.04 μM, respectively, which was comparable to that in BaF3 parental cells with IC_50_ of 1.31 μM (Figure 2A), indicating the weak cytotoxic effect of HCD3514.

Thus, these results demonstrated the *in vitro* anti-tumor activity of HCD3514 in EGFR^T790M/C797S^ triple mutant cells through suppression of EGFR phosphorylation, leading to the inhibition of cellular proliferation.

### HCD3514 induced apoptosis in cells harboring EGFR^C797S^ triple mutation

**Figure 3:**
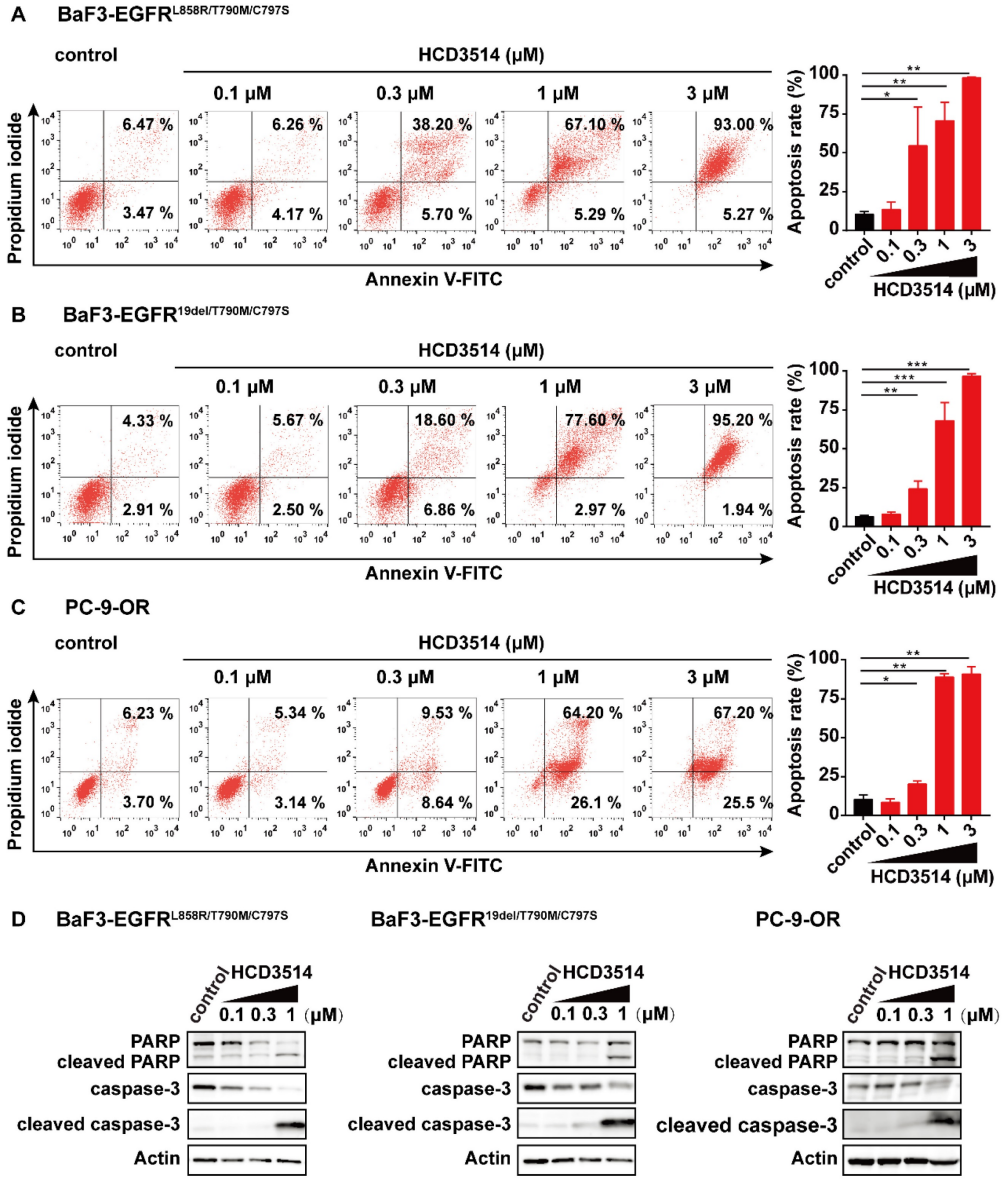
** The apoptosis-inducing effect of HCD3514 in EGFR^C797S^ triple mutant cells.** HCD3514 induced apoptosis in BaF3-EGFR^L858R/T790M/C797S^ cells **(A)**, BaF3-EGFR^19del/T790M/C797S^ cells **(B)** and PC-9-OR cells **(C)**, and representative experiments were shown. Quantitative analyses of apoptosis rates were depicted and experiments were caried out for three times. **(D)** The expression of apoptosis-related proteins, including PARP, cleaved PARP, caspase-3 and cleaved caspase-3 were detected by Western blot analysis. *P < 0.05, **P < 0.01, *** P < 0.001.

Given that NSCLC cells harboring EGFR mutations have been reported to undergo apoptosis 41, 42, we assessed the effect of HCD3514 treatment on cell apoptosis. As shown in Figure 3, the proportion of apoptotic cells in HCD3514-treated cells increased in a dose-dependent manner compared to vehicle-treated cells. In BaF3-EGFR^L858R/T790M/C797S^ cells, HCD3514 triggered significant apoptosis with apoptosis rates of 72.39% and 98.27% at the concentration of 1 μM and 3 μM, respectively (Figure 3A), similar results were detected in BaF3-EGFR^19del/T790M/C797S^ cells (Figure 3B) and PC-9-OR cells (Figure 3C). In contrast, brigatinib only induced significant apoptosis at the concentration of 3 μM with apoptosis rates around 80% in all three EGFR^C797S^ mutant cells, indicating that HCD3514 exhibited more potent apoptosis-inducing efficacy than brigatinib (Supplementary Figure S2).

To gain deeper insight into the potential mechanism of apoptosis induction in EGFR^C797S^ triple mutant cells, the expressions of apoptosis-related genes were tested by Western blot analysis. Poly ADP-ribose polymerase (PARP) and cysteinyl aspartate specific proteinase-3 (caspase-3) play an important role in cellular processes, including apoptosis 43, 44. It was observed that the expression of caspase-3 was down-regulated after treatment with HCD3514 in triple mutant BaF3 and PC-9-OR cells. Correspondingly, the expressions of pro-apoptotic proteins, including cleaved caspase-3 and cleaved PARP were dose-dependently up-regulated (Figure 3D). In conclusion, HCD3514 induced apoptosis through a caspase-related mechanism.

### HCD3514 suppressed tumor growth in EGFR^C797S^ triple mutant BaF3 xenograft model

**Figure 4:**
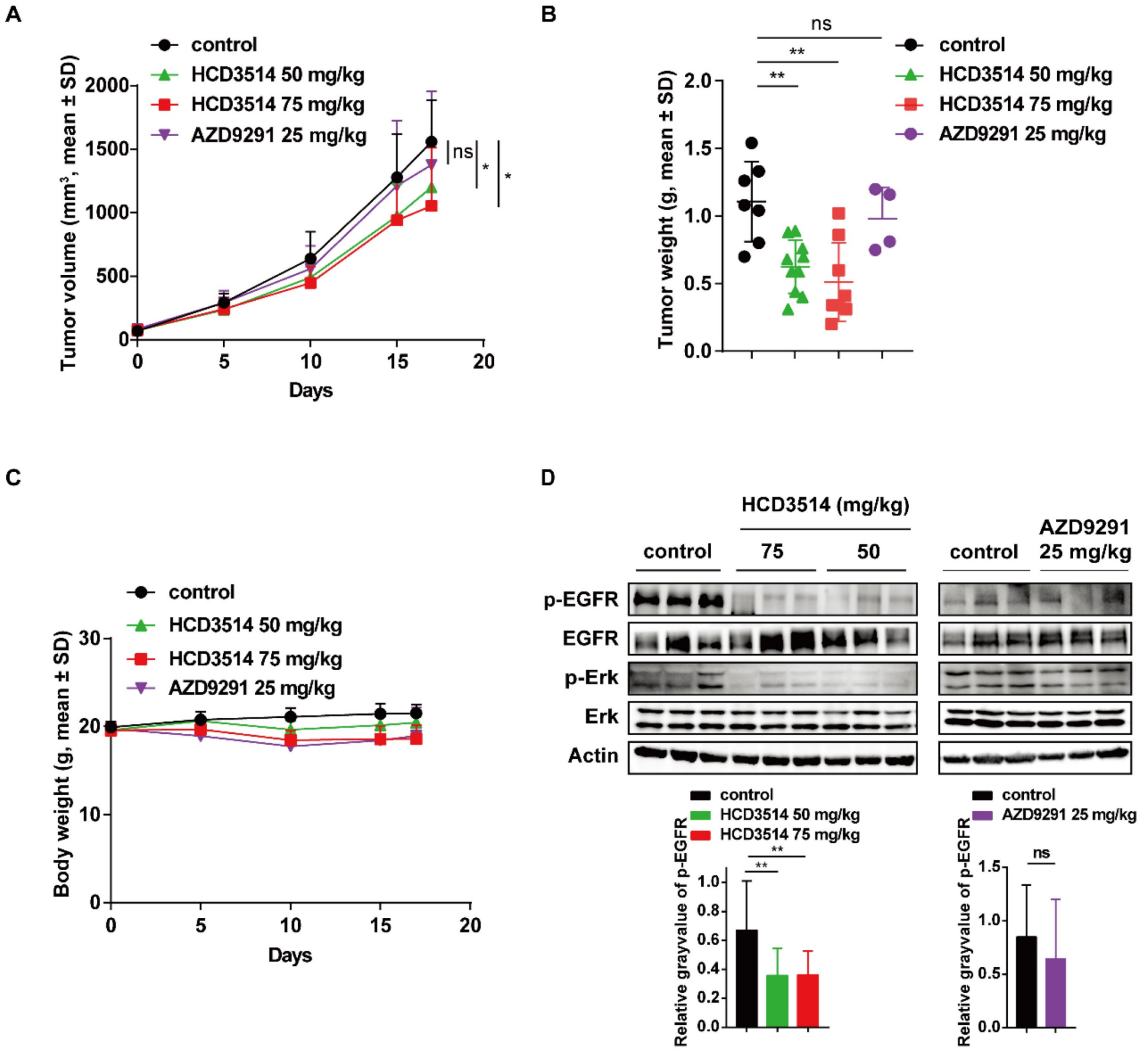
** Antitumor activity of HCD3514 in EGFR^19del/T790M/C797S^ mutant BaF3 xenograft model. (A)** Mice bearing EGFR^19del/T790M/C797S^ mutant tumors were orally treated with HCD3514 at the concentration of 50, 75 mg/kg and AZD9291 at the concentration of 25 mg/kg. Tumor volumes were measured once per week and presented as mean ± SD. **(B)** Two hours later after oral administration vehicle, HCD3514 or AZD9291 in mice bearing EGFR^19del/T790M/C797S^ BaF3 cells, tumor tissues were harvested and tumor weight were measured. **(C)** Body weight for each dose were measured. **(D)** The inhibitory activity of phospho-EGFR and phospho-Erk in tumors was assessed by Western blot analysis, with actin as a control. *P < 0.05, **P < 0.01.

Based on the high potency of HCD3514, we then evaluated the activity of HCD3514 *in vivo* in BaF3-EGFR^19del/T790M/C797S^ xenograft mouse model. BALB/c nude mice bearing established BaF3-EGFR^19del/T790M/C797S^ mouse xenograft tumors were daily oral treatment with HCD3514 (50 and 75 mg/kg), AZD9291 (25 mg/kg) or vehicle control. Consequently, HCD3514 resulted in an obvious growth inhibition of BaF3-EGFR^19del/T790M/C797S^ mouse xenograft tumors in a dose-dependently manner with tumor growth inhibition (TGI) values of 22.53% and 37.73% at the dosage of 50 mg/kg and 75 mg/kg, respectively, while AZD9291 treatment did not show significant reduction of tumor growth in the xenograft model compared to the vehicle treated mice (Figure 4A). Then tumor tissues were collected for follow-up detection after 2 h of the last treatment with HCD3514, AZD9291 or vehicle. Tumor weight was reduced with the increased concentration of HCD3514 (Figure 4B). In addition, the treatment was well-tolerated, with body weight maintained throughout the study, suggesting a favorable safety of HCD3514 (Figure 4C).

The antitumor potency of HCD3514 in BaF3-EGFR^19del/T790M/C797S^ mouse xenograft model was further confirmed by Western blotting that showed decreased phosphorylation of EGFR and the downstream signaling phosphorylation of Erk level, indicating that the tumor growth inhibition of the resistant triple mutant model due to the inhibition of the activation of EGFR and EGFR-related molecule. However, as expected, AZD9291 treatment showed no obvious inhibitory activities of EGFR and Erk (Figure 4D).

Taken together, we identified HCD3514 as a potent and orally active fourth-generation EGFR inhibitor and exhibited an excellent antitumor effect *in vitro* and* in vivo*.

### HCD3514 inhibits not only EGFR^T790M/C797S^ triple mutations, but also EGFR^T790M^ double mutations

**Figure 5:**
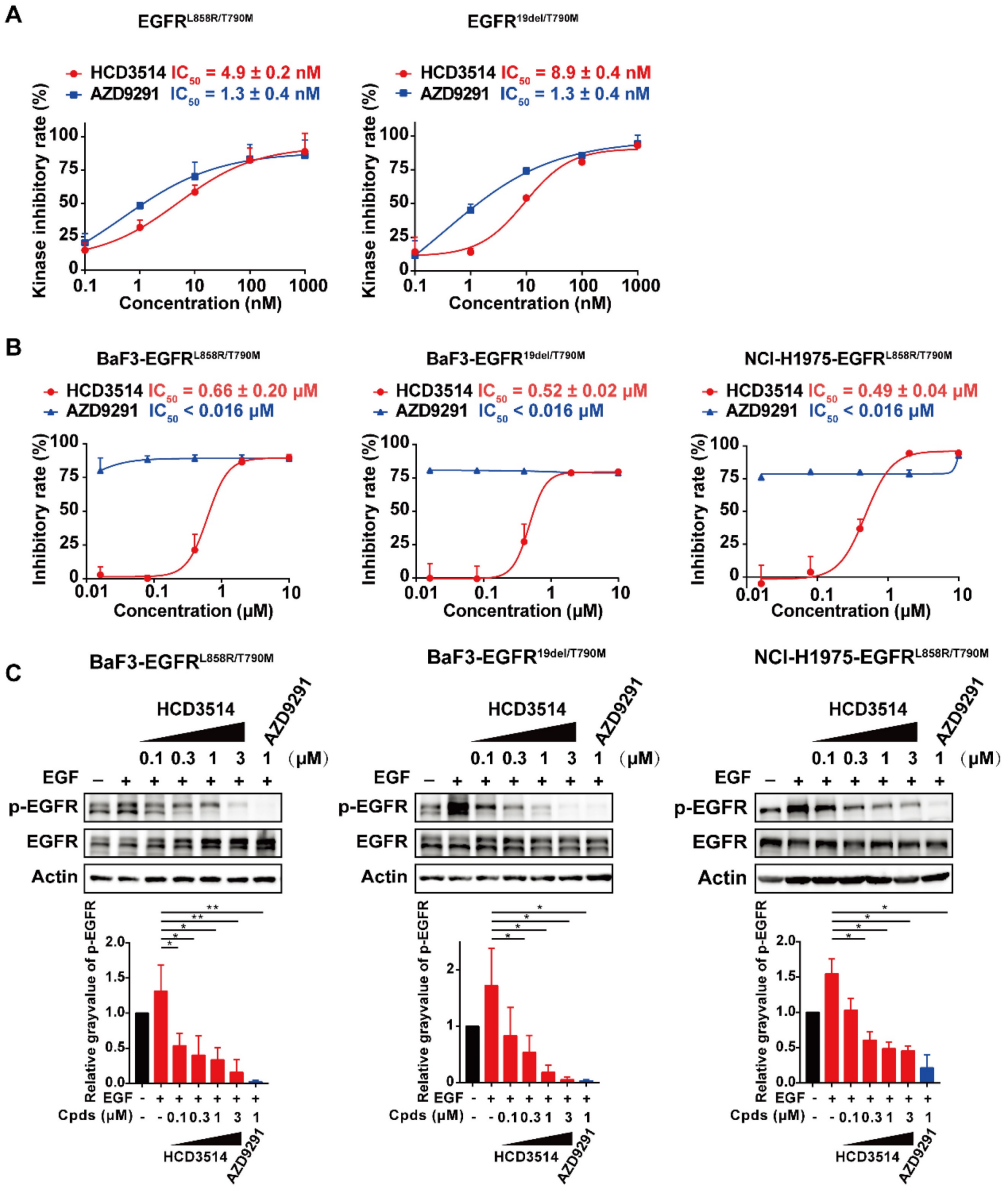
** Efficacy of HCD3514 against EGFR^T790M^ double mutations. (A)** Kinase inhibitory activities of HCD3514 and AZD9291 against EGFR^L858R/T790M^ and EGFR^19del/T790M^. The IC_50_ values were shown as mean ± SD of three independent experiments. **(B)** Antiproliferation activity of HCD3514 and AZD9291 in BaF3 cells that overexpressed EGFR^L858R/T790M^ or EGFR^19del /T790M^ double mutation, as well as NCI-H1975 cells. The IC_50_ values were shown as mean ± SD of three independent experiments. **(C)** The activities of the compounds on the activation of EGFR mutant in EGFR^T790M^ double mutant cells. All cells were treated with HCD3514 or AZD9291 at indicated concentration for 2 h, and the expression of phospho-EGFR and total EGFR was detected. The phospho-EGFR expression was quantified by ImageJ software and data shown as mean ± SD of three independent experiments. *P < 0.05, **P < 0.01.

In addition, we checked whether HCD3514 inhibits EGFR-double mutations (EGFR^L858R/T790M^ and EGFR^19del/T790M^), as suggested by the molecular docking experiment showed in Figure 1C. According to the docking results, the aminopyrimidine core of HCD3514 formed a bidentate hydrogen bond interaction with the “hinge” residue Met793 and the chloride atom was directed toward the mutated Met790. The hydrophobicity conferred by Met790 mutation likely contributes to the potency of HCD3514 against the T790M mutant. As shown in Figure 5, HCD3514 potently inhibited EGFR^L858R/T790M^ and EGFR^19del/T790M^ mutations with IC_50_ values of 4.9 and 8.9 nM in biochemical, respectively. Moreover, HCD3514 showed antiproliferative activities in BaF3-EGFR^L858R/T790M^ and BaF3-EGFR^19del/T790M^ cells with IC_50_ of 0.66 μM and 0.52 μM, respectively, although the IC_50_ values of HCD3514 were higher than that of AZD9291. Furthermore, Western blot analysis revealed that HCD3514 dose-dependently suppressed the EGFR phosphorylation in both BaF3-EGFR^L858R/T790M^ cells and BaF3-EGFR^19del/T790M^ cells. Besides, we also evaluated the *in vitro* anti-tumor activity of HCD3514 in NCI-H1975 cells harboring EGFR^L858R/T790M^. HCD3514 also displayed an excellent antiproliferative potency in NCI-H1975 cells with IC_50_ of 0.49 μM and the expression of phospho-EGFR was down-regulated after treatment with HCD3514 in a dose-dependent manner. Thus, HCD3514 could target EGFR^T790M^ double mutations as well as EGFR^T790M/C797S^ triple mutations.

## Discussion

Oncogenic mutations in EGFR are prevalent in NSCLC. Though third-generation EGFR inhibitor AZD9291 has achieved a great clinical benefit in NSCLC patients, the EGFR^C797S^ acquired mutation would compromise the therapeutic efficacy. Currently, no approved small molecule EGFR inhibitors are available for NSCLC patients with resistant triple mutant EGFR, and thus, discovery of a potent and high selective EGFR inhibitor to overcome the AZD9291 mediated resistance was urgent and meaningful. Current discovery strategies of the fourth-generation EGFR inhibitors include: 1) exploiting allosteric binding pocket by allosteric inhibitors. 2) developing ATP-competitive inhibitors by structural modification of ALK/EGFR inhibitor brigatinib or the third-generation EGFR TKIs.

In this study, through structural hybridization of brigatinib with EGFR^T790M^ inhibitor AZD9291, we identified a new fourth-generation EGFR triple mutant-selective inhibitor HCD3514, which possessed a promising antitumor activity both* in vitro* and *in vivo*. Compared to AZD9291, molecular docking of HCD3514 identified an additional hydrogen bond interaction towards Lys745, which was proposed to compensate for the loss of covalent bound due to the C797S point mutation and ultimately enhanced the inhibitory efficacy of HCD3514 on EGFR^C797S^ mutations. HCD3514 displayed a single digit-nanomolar kinase inhibitory activity against triple mutant EGFR, including EGFR^L858R/T790M/C797S^ and EGFR^19del/T790M/C797S^, which showed superior biochemical activity than brigatinib. Importantly, HCD3514 exhibited a sufficient EGFR^WT^ sparing window, suggesting its low toxicity. In addition, HCD3514 simultaneously inhibited the activities of both mutant forms of EGFR at the cellular level. It showed that both engineered BaF3 cells harboring EGFR^L858R/T790M/C797S^ or EGFR^19del/T790M/C797S^ were sensitive to HCD3514, as well as the PC-9-OR cells (EGFR^19del/T790M/C797S^ expressing PC-9 lung cancer cells), with enhanced inhibition the phosphorylation of EGFR. Besides, cleaved PARP and cleaved caspase-3 levels were showed to increase in three C797S mutant cells, which was consistent with apoptosis induction in a concentration-dependent manner. Furthermore, unlike EAI045 or brigatinib which showed limited antitumor effect without combined with anti-EGFR antibody 24, 26, HCD3514 as a single agent exhibited potent antitumor efficacy in tumor xenograft model in a dose-dependent manner without toxicity suggested the potential to target C797S mutant tumors. HCD3514 treatment revealed a significant inhibition of phosphorylation of EGFR in tumor xenografts, supporting that the antitumor efficacy of HCD3514 was through the inhibition of AZD9291-resistant EGFR mutations. Moreover, HCD3514 potently inhibited not only EGFR^C797S^ triple mutations but also EGFR^T790M^ double mutations.

In summary, our preclinical data demonstrated that HCD3514 was effective in overcoming both triple mutant and double mutant forms of EGFR-dependent resistance mechanisms *in vitro* and* in vivo* and we also provided a novel chemical scaffold and a new lead compound that worthy of further investigation.

## Supplementary Material

Supplementary figures.

## Abbreviations

EGFR: generation epidermal growth factor receptor
TKI: tyrosine kinase inhibitor
NSCLC: non-small cell lung cancer
EGFR^WT^: wild-type EGFR
19del: the small in-frame deletions in exon 19
DSMZ: Deutsche Sammlung von Mikroorganismen und Zellkulturen GmbH
ECACC: European Collection of Authenticated Cell Cultures
SP: standard precision
CCK8: cell counting kit-8
SRB: sulforhodamine B
TGI: tumor growth inhibition
SD: Standard Deviation
PARP: Poly ADP-ribose polymerase
caspase-3: Cysteinyl aspartate specific proteinase-3
