# Supplementary material for: Discovery of HCD3514 as a potent EGFR inhibitor against C797S mutation in vitro and in vivo
Source: J Cancer. 2023 Jan 1;14(1):152–62. doi: 10.7150/jca.77788 (PMC9809337; doi:10.7150/jca.77788)
Supplement: Supplementary file 1 — Supplementary figures. [file jcav14p0152s1.pdf]

Supplementary material

Discovery of HCD3514 as a potent EGFR inhibitor against C797S mutation *in vitro* and *in vivo*

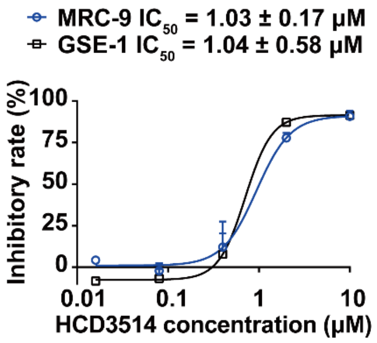

Supplementary Figure S1. Antiproliferative activities of HCD3514 in two human normal cell lines (MRC-9 cells and GSE-1 cells). The IC_{50} values were shown as mean ± SD of three independent experiments.

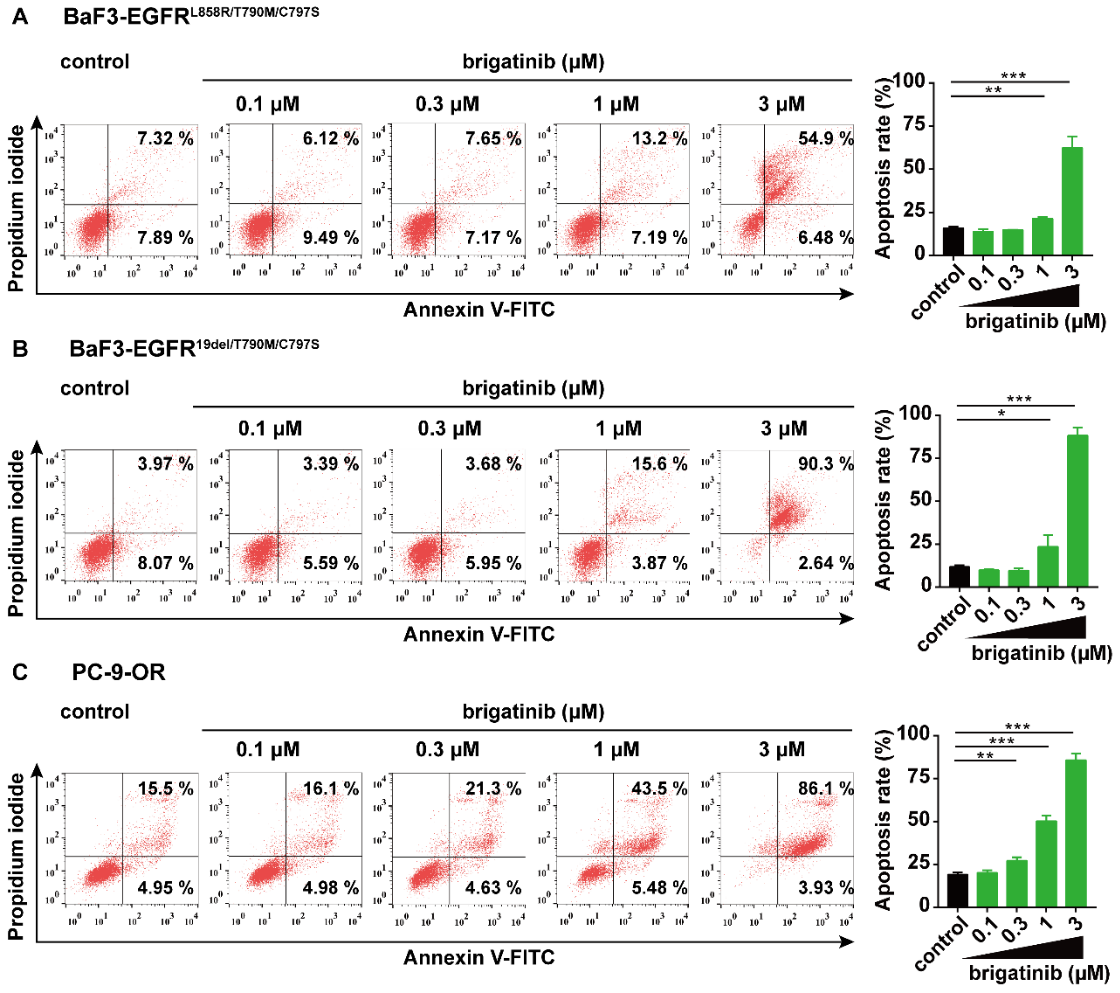

Supplementary Figure S2. The apoptosis-inducing effect of brigatinib in EGFR^{C797S} triple mutant cells. HCD3514 induced apoptosis in BaF3-EGFR$^{L858R/T790M/C797S}$ cells (A), BaF3-EGFR$^{19del/T790M/C797S}$ cells (B) and PC-9-OR cells (C), and representative experiments were shown. Quantitative analyses of apoptosis rates were depicted and experiments were caried out for three times.
